# Modularity of a leaf moth-wing pattern and a versatile characteristic of the wing-pattern ground plan

**DOI:** 10.1186/1471-2148-13-158

**Published:** 2013-07-27

**Authors:** Takao K Suzuki

**Affiliations:** 1 Laboratory for Evolutionary Morphology, Center for Developmental Biology, RIKEN, 2-2-3 Minatojima-minami, 650-0047 Chuo-ku Kobe, Japan; 2 Current address: Transgenic Silkworm Research Unit, Genetically Modified Organism Research Center, National Institute of Agrobiological Sciences, 1-2 Owashi, 305-8634 Tsukuba, Japan

**Keywords:** Morphological integration, Modularity, Evolvability, Moth and butterfly wing patterns, Masquerade, Leaf mimicry, Nymphalid ground plan, Geometric morphometrics, Correlation network

## Abstract

**Background:**

One of the most intriguing questions in evolutionary developmental biology is how an insect acquires a mimicry pattern within its body parts. A striking example of pattern mimicry is found in the pattern diversity of moth and butterfly wings, which is thought to evolve from preexisting elements illustrated by the nymphalid ground plan (NGP). Previous studies demonstrated that individuality of the NGP facilitates the decoupling of associated common elements, leading to divergence. In contrast, recent studies on the concept of modularity have argued the importance of a combination of coupling and decoupling of the constituent elements. Here, we examine the modularity of a mimicry wing pattern in a moth and explore an evolvable characteristic of the NGP.

**Results:**

This study examined the wings of the noctuid moth *Oraesia excavata*, which closely resemble leaves with a leaf venation pattern. Based on a comparative morphological procedure, we found that this leaf pattern was formed by the NGP common elements. Using geometric morphometrics combined with network analysis, we found that each of the modules in the leaf pattern integrates the constituent components of the leaf venation pattern (i.e., the main and lateral veins). Moreover, the detected modules were established by coupling different common elements and decoupling even a single element into different modules. The modules of the *O*. *excavata* wing pattern were associated with leaf mimicry, not with the individuality of the NGP common elements. For comparison, we also investigated the modularity of a nonmimetic pattern in the noctuid moth *Thyas juno*. Quantitative analysis demonstrated that the modules of the *T*. *juno* wing pattern regularly corresponded to the individuality of the NGP common elements, unlike those in the *O*. *excavata* wing pattern.

**Conclusions:**

This study provides the first evidence for modularity in a leaf mimicry pattern. The results suggest that the evolution of this pattern involves coupling and decoupling processes to originate these modules, free from the individuality of the NGP system. We propose that this evolution has been facilitated by a versatile characteristic of the NGP, allowing the association of freely modifiable subordinate common elements to make modules.

## Background

**Figure 1:**
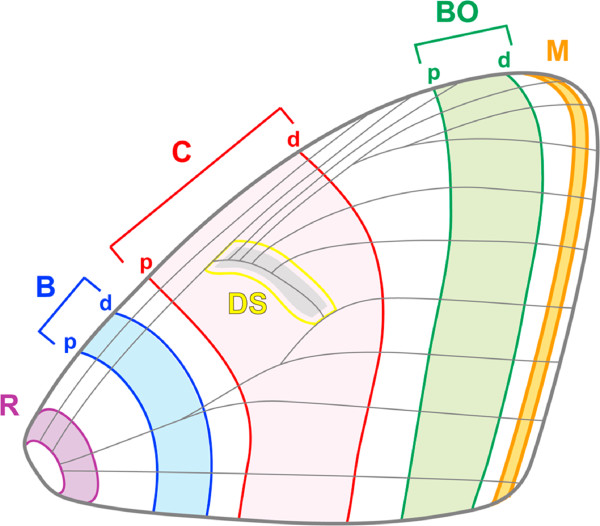
**Nymphalid ground plan.** The ground plan of moth wing patterns. This scheme consists of 10 elements including 3 symmetry pattern elements (the proximal (p) and distal (d) bands), designated as basal (B; blue), central (C; red), and border (BO; green) elements, and 4 elements designated as root (R; purple), submarginal and marginal (M; orange), and discal spot (DS; yellow) elements.

Understanding how animal body parts are structured to perform their function is crucial for understanding morphological divergence and adaptation in animal evolution [1-7]. A valuable clue to understanding the diversification of animal structure comes from a comparative morphological perspective showing that new morphology rarely arises from *de novo* body parts, but rather emerges from preexisting parts with lineage-specific modifications [8-12]. Mammalian skeletal limbs are a well-known illustration of this point, wherein diversified skeletal limb structures (e.g., bat wings, whale flippers, and human hands) are composed of a common set of skeletal parts (e.g., humerus, ulna, radius, and digits) [13,14]. Such divergence is also found in the spectacular diversity of moth and butterfly wing patterns, which are thought to result from a common set of symmetry pattern elements shared across numerous species (termed the nymphalid ground plan; NGP) [15-17] (Figure 1). Recently, the establishment of the NGP was also supported by molecular experimental data showing that some elements of the NGP are regulated by the gene expression of a well-known morphogen, *wnt-1*, in different families (e.g., noctuid moths and nymphalid butterflies) [18,19]. Thus, the NGP provides a comprehensive framework for identifying diversified wing patterns as morphological structures composed of a common set of pattern elements.

Pattern divergence in moth and butterfly wings has occurred via modifications in the association between pattern elements. Previous studies have suggested that each symmetry element of the NGP appears to be genetically and phenotypically autonomous [20-23] and can become developmentally decoupled [17,24-26], thereby allowing separate evolutionary trajectories. Several lines of experimental evidence suggest that the high individuality of the NGP allows further decoupling of the pattern elements (e.g., dislocation), and that this characteristic contributes to the evolvability of lepidopteran wing patterns [17,24-26]. In contrast, recent studies on morphological integration have emphasized the importance of a combination of coupling and decoupling of body part development for adaptation of animal structures [3,27,28]. The concept of morphological integration postulates that functionally related elements are tightly coupled, whereas unrelated parts are independently decoupled [3,27,28]. According to this concept, it seems to be hypothesized that a specific integrated nature can be detected in lepidopteran wing patterns, in particular, complex adaptive patterns such as leaf mimicry. Thus, to understand how a lepidopteran wing pattern uses its subordinate elements, investigation of the integrated nature (i.e., coupling and decoupling) of the NGP symmetry elements for a complex adaptive wing pattern is necessary.

**Figure 2:**
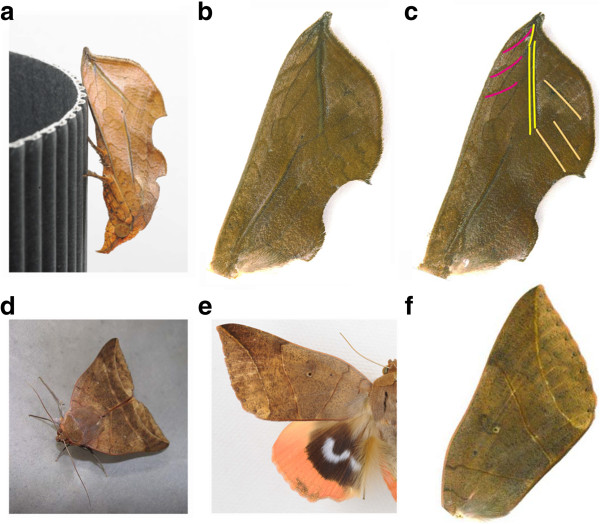
**Camouflage patterns on two moth wings.** These two moths belong to the same family, Noctuidae, but display different types of wing patterns. **(a)** When resting, O. excavata displays a leaf pattern to potential predators. **(b)** The male dorsal right forewing has an outline that resembles a leaf. **(c)** The forewing mimics leaf venation, comprising a main vein and right and left lateral veins (highlighted with yellow, orange, and pink lines, respectively). **(d)** When resting, the forewings of T. juno fold horizontally. **(e)** The dorsal wings of a male moth. **(f)** The forewing pattern consists of four parallel lines that result in a chevron-shaped mark in the folded wings.

The pre-eminent model of complex adaptive patterns is leaf mimicry in moths, as most of their patterns are composed of multiple parts visually arranged to look like leaves. One of the most striking examples of a leaf moth is the Japanese noctuid moth *Oraesia excavata*, whose dorsal forewings exhibit a special resemblance to a leaf with leaf venation patterns [29] (Figure 2a-c). At rest, the moth remains still and sports a leaf-shaped outline of the forewings; such behavior and shape strongly support their mimesis to leaves. Additionally, if their morphological integration is indispensable to the leaf mimicry pattern, the specificity of its integrated nature could be highlighted by comparison with the nonmimetic patterns of another moth. To test this approach, we examined the wing pattern of another type of moth, *Thyas juno*, which displays a relatively simple pattern (Figure 2d-f). The leaf venation pattern of *O*. *excavata* and the nonmimetic pattern of *T*. *juno* are composed of several pigmental elements (not a venous pattern; see Additional file 1), providing a suitable model for exploring the integrated nature of wing patterns.

A key feature to characterize the integrated nature of complex wing patterns is provided by modularity, which describes tightly coupled units and individually decoupled units [28,30-32]. Modularity represents developmental and functional aspects of morphological structures, since it arises from developmental interactions [31,33] and is shaped by the accumulated effects of natural selection [28,34,35]. Although the field of network theory [36,37] has significantly advanced the ability to detect modules, it has been minimally applied to deciphering the integration and modularity of morphological structures. To achieve this, one must develop a new method to apply a network theoretical approach to multivariate correlation data. A “correlation network” [38,39] approach has recently been developed whereby the nodes represent constituent elements such as metabolites, neurons, or genes, and the links represent the correlation of a characteristic of the elements such as metabolic flux, neuronal activity, or gene expression above a threshold level. This method has been explicitly, or at least implicitly, employed in various studies on metabolomics [38-40], neurodynamics [41], and transcriptomes based on gene co-expression [42,43]. Likewise, this method can be applied to explore the modules of morphological structures (termed morphological correlation network), whereby the nodes represent the constituent parts and the links represent the correlation of the spatial positions among them.

Here, we examine whether leaf and nonmimetic wing patterns are subject to the NGP. Additionally, we quantified the modules of these wing patterns and investigated how these modules couple and decouple the NGP symmetry elements. Based on the comparative morphological analysis, we dissected both the *O*. *excavata* leaf pattern and the nonmimetic pattern into a common set of NGP symmetry elements. Subsequently, using a morphological correlation network, quantitative analysis showed that the modules of the *T*. *juno* wing pattern regularly corresponded to the individuality of the NGP symmetry elements. Furthermore, quantitative analysis detected the modules of the leaf pattern, each of which corresponded to a component of the leaf venation pattern (i.e., the main and lateral venation patterns). Unlike the *T*. *juno* wing pattern, the modules of the *O*. *excavata* wing pattern were closely associated with leaf mimesis, not to the individuality of the NGP symmetry elements. The results indicate that the modules detected in the leaf mimicry pattern are established by a combination of coupling and decoupling the NGP symmetry elements.

## Results

### Groundplan-based dissection of the leaf wing pattern

**Figure 3:**
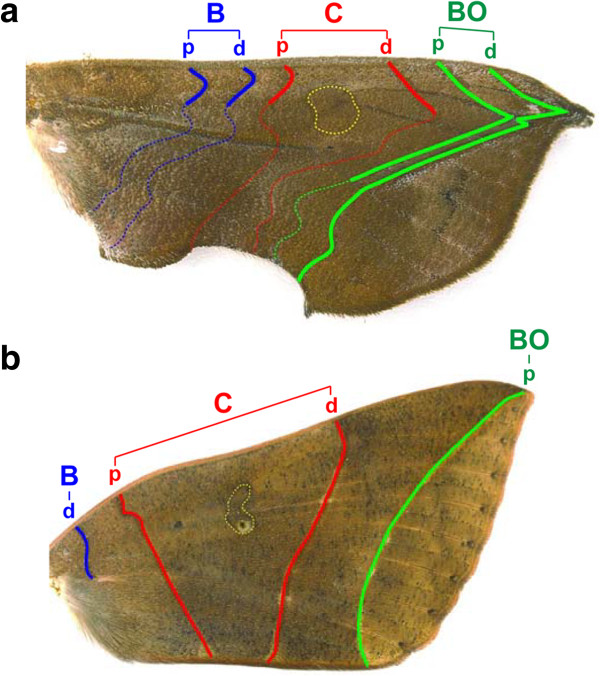
**Ground plan and the modification towards camouflage patterns. (a)** Ground plan identified in the dorsal right forewing of an O. excavata male. According to this scheme, the leaf pattern can be dissected into a set of NGP symmetry elements. **(b)** Ground plan identified in the dorsal right forewing of a T. juno male. Accordingly, these two moth wings share the homologous elements of the symmetry systems (B, C, and BO). The NGP symmetry elements are illustrated by the same colors as in Figure 1.

It has been proposed that the scheme of the NGP (Figure 1) is established in diversified moth wing patterns in various families (see review in references [17,44] including the Noctuidae family [44,45]). To examine whether the *O*. *excavata* and *T*. *juno* wing patterns are subject to the NGP, we investigated which pigmental elements of the wing patterns correspond to which symmetry elements (B, C, and BO) constituting the NGP (Figure 3). Comparative morphological analysis dissected the *O*. *excavata* leaf venation pattern into a set of NGP symmetry elements (Figure 3a): the left lateral vein was composed of the left ends of Cd, BOp, and BOd; the main vein was composed of the central portions of BOp and BOd; and the right lateral vein was composed of the right extremities of BOd and M (Figures 2c and 3a). According to this analysis, the outward appearance of the leaf pattern is achieved by elaborate modification of the geometrical shapes of the elements. For example, Cd, BOp, and BOd are formed in an acute angle; BOd is formed in an obtuse angle; the upper-side parts of Cd, BOp and BOd are arranged in parallel; and the middle parts of BOp and BOd are present in straight lines arranged in parallel. In summary, the leaf pattern in *O*. *excavata* is captured as a derivative of the NGP. For comparison, we examined the wing pattern of *T*. *juno*, another noctuid moth from the same family. The wing pattern in *T*. *juno* does not display leaf venation patterning, but instead consists of four parts demarcated by almost straight lines (Figure 2d-f). Similarly, this wing pattern can be traced as a set of elements: Bd, Cp, Cd, and BOp (Figure 3b). Although their appearances are largely different, the scheme of the ground plan suggests that these two moths are composed of homologous elements inherited from a common ancestor. Compared to the *T*. *juno* wing pattern, the *O*. *excavata* wing pattern seems to have more elaborate modifications (Figure 3).

### The modules detected in the O. excavata leaf pattern

**Figure 4:**
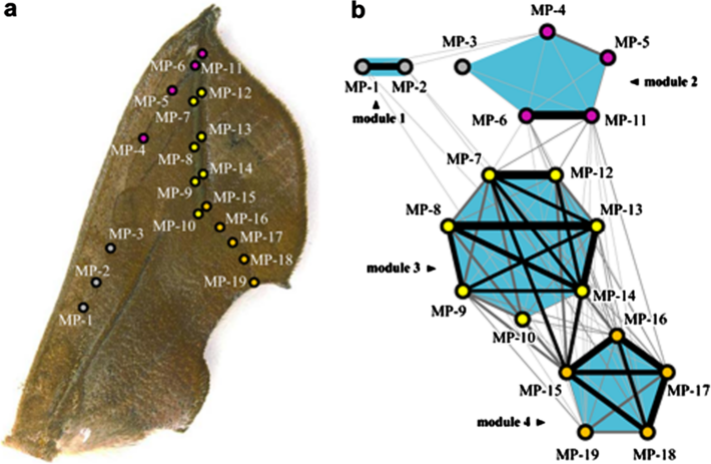
**Morphological correlation network in the O. excavata wing pattern. (a)** Measurement points were set on the O. excavata leaf wing pattern. **(b)** The correlations among the measurement points were quantified and visualized as a form of network. In this correlation network, the nodes represent the measurement points, whereby the links represent correlations between the measurement points above the threshold level (Rv coefficient = 0.2). In this correlation network, the RB method detected four modules (light blue areas). Because the RB method finds weighted solutions, the most frequent solution (67.6% occurrence frequency of the 10,000 trials) is shown. Larger correlation coefficients are shown by thicker edge widths and blacker coloration of links. The measurement points within modules are shown in the same colors as the measurement points in Figure 4a.

Quantitative analysis with high-resolution measurements of the variation at a single pigment-cell level (Additional file 2) elucidated the correlation network of the *O*. *excavata* wing pattern (Figure 4). To draw this network, all possible combinations of pairwise correlations between the measurement-point set on the wing pattern were calculated (Additional file 3). Correlational relationships above the threshold level (Rv coefficient > 0.2) were visualized in the form of a correlation network [38,39], from which the Reichardt-Bornholdt (RB) method [46] succeeded in extracting four modules (Figure 4b; Additional file 4e-h). Because this method finds weighted solutions even in the same data set, the resultant modular architectures are represented with occurrence frequencies in repeated trials (see Methods). Although the repeated trials seeking solutions found a different set of modules in each of the trials, each complete module in the most frequent solution was detected with high reproducibility: 74.5% of the 10,000 trials for module 1, 70.3% of the trials for module 2, 98.2% of the trials for module 3, and 97.9% of the trials for module 4 (Additional file 4e-h). Subsequently, we tested whether the modules detected from the correlation network (threshold level of the Rv coefficient = 0.2) could be changed with respect to changes in the threshold levels (threshold level of the Rv coefficient = 0.4 and no threshold). Because the topology of the correlation network largely depends on the choice of the threshold level [40], the modules detected by the RB method have the possibility of being sensitive to the choice of the threshold. Despite this possibility, the test analysis confirmed that the modules detected from the standard correlation network were robust regardless of the choice of threshold level (no threshold, Additional file 4a-d; threshold level of Rv coefficient = 0.4, Additional file 4i-j). In particular, the most frequent (49%) and the second most frequent (38%) modular architectures were exactly the same as shown in the correlation network (Rv coeff. threshold level = 0.2), except for the frequencies of the modular architectures (Additional file 4a-d). Taken together, we conclude that the leaf pattern in *O*. *excavata* consists of four modules.

### The modules detected in the T. juno nonmimetic pattern

**Figure 5:**
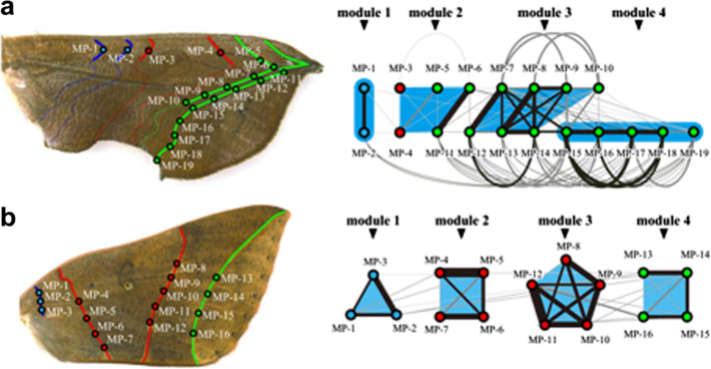
**Comparison between the correlation networks of the leaf and nonmimetic patterns.** From the correlation networks, the RB method detected modules (light blue areas). **(a)** Correlation network (Rv coeff. threshold level = 0.2) of the O. excavata wing pattern is re-plotted based on the ground plan. The detected modules correspond to the NGP symmetry elements in a complex manner. **(b)** Correlation network (Rv coeff. threshold level = 0.2) of the T. juno wing pattern is shown on the ground plan. The detected modules regularly correspond to the NGP symmetry elements in a one-to-one manner. The constituent elements of the wing patterns and the measurement points are represented by the same colors as in Figure 3. Larger correlation coefficients are shown by thicker edge widths and blacker coloration of links.

The morphometrical analysis using the RB method quantified the correlation network of the *T*. *juno* wing pattern, which was found to be composed of four modules (Figure 5b). The repeated trials seeking solutions showed a high reproducibility for all of the modules: module 1 (MP1-MP3), 92.4% occurrence frequency in 10,000 trials; module 2 (MP4-MP7), 96.9%; module 3 (MP8-MP12), 99.9%; and module 4 (MP13-MP16), 92.5%. In addition, we validated that the modules detected in the correlation networks (threshold level of the Rv coefficient = 0.2) were robust with respect to changes in the threshold levels (threshold level of the Rv coefficient = 0.4 and no threshold). These results suggest that the nonmimetic pattern in *T*. *juno* consists of four modules.

### Module construction of *O*. *excavata* wing pattern is associated with leaf mimesis

The identified modules in the *O*. *excavata* wing pattern are closely associated with the leaf venation pattern (Figure 4). Among the four modules, three correspond approximately with each component of the leaf venation pattern: module 2 corresponds to the left lateral vein, module 3 corresponds to the main vein, and module 4 corresponds to the right lateral vein.

To explore how the modules detected in the leaf pattern are constructed by the NGP symmetry elements, the correlation network of the *O*. *excavata* wing pattern was re-plotted on the basis of the NGP (Figure 5a). Module 1 was composed of the measurement points (MP1, MP2) located on Bp and Bd. This module regularly corresponded to the B symmetry system in a one-to-one manner, suggesting that the construction of this module is consistent with previous studies [21-23]. In contrast, modules 2-4 did not correspond to the symmetry systems in a one-to-one manner, but in a more complex fashion (Figure 5a). Module 2 was composed of the measurement points belonging to two different symmetry systems (MP3 and MP4 located on the C symmetry system; MP5, MP6, and MP11 located on the BO), thus coupling the upper side of the Cd and BOp. Module 3 was composed of the measurement points MP7-MP10 and MP12-MP14, and module 4 was composed of MP15-MP19, with each module partially corresponding to the BO symmetry system. These results suggest that modules 2, 3, and 4 originated by decoupling the BO symmetry elements into separately correlated units, although the elements remained as continuous lines. Taken together, the elements constituting the *O*. *excavata* wing pattern were modularized as tightly correlated units, regardless of the units in the NGP symmetry systems, although the NGP remained identifiable.

Finally, we investigated how the modules in the *T*. *juno* wing pattern were associated with the NGP. Module 1 corresponded to the Bd element, module 2 corresponded to the Cp element, module 3 corresponded to the Cd element, and module 4 corresponded to the BOp element (Figure 5b). In contrast to the complicated establishment of the modules in the *O*. *excavata* wing pattern, all of the modules of the *T*. *juno* wing pattern regularly corresponded to the NGP symmetry systems in a one-to-one manner, whereby no module coupled elements derived from a different NGP symmetry system nor decoupled a single element into separately correlated units.

## Discussion

Using quantitative analysis, this study clearly provides the first evidence for modularity in a leaf mimicry pattern. We have shown that the modules detected were established by coupling different symmetry elements and decoupling even a single element into different modules. Moreover, the modules of the *O*. *excavata* wing pattern were closely associated with leaf mimicry, not to the individuality of the NGP symmetry elements. For comparison, we also investigated the integrated nature of the nonmimetic wing pattern of *T*. *juno*. Unlike the *O*. *excavata* wing pattern, quantitative analysis of the *T*. *juno* wing pattern demonstrated that the modules regularly corresponded to the individual NGP symmetry elements. These results suggest that the evolution of the leaf mimicry pattern entails the evolution of new modules, free from the individuality of the NGP symmetry system.

Recent attempts to identify developmental modules have advocated that two spatially adjacent measures are likely to have a higher correlation than more distant measures ([47,48]; see also [49,50] for Pearson’s rule). Adjacency within pigmental patterns in butterfly and moth wings seems to reflect actual developmental processes, given that the pigmental elements are more or less directly formed by morphogen diffusion mechanisms [17-19,24,51,52]. Therefore, we are confident that our results reflect the underlying biological mechanisms (i.e., the developmental processes and the accumulated consequences for adaptation), because spatial autocorrelation cannot account for the entire pattern revealed in our analyses. For example, we found a degree of independence between the adjacent B and C symmetry systems in the *O*. *excavata* wing pattern (Figure 5a): MP2 (B) and MP3 (C) were adjacently located (distance = 240 units) but showed no significant correlation, whereas MP1 (B) and MP2 (B) were located at nearly the same distance (192 units) and showed high correlation (Rv coefficient = 0.71). Additionally, we found phenotypic independence within the BO symmetry system; although MP5-MP19 were adjacently located (all points comprised the BO symmetry system), some points were tightly modularized, with some adjacent points decoupled (probably due to developmental compartments of wing veins) (Figure 5a).

Our understanding of conceptual issues (such as modularity) is tightly linked to progress in the quantification methods used to detect such issues. Methods of detecting modular architectures in organismal bodies have been well developed [47,53-55] and these approaches have been applied to various organismal bodies: for example, cichlid jaws [56], monkey dentition [57], hominoid crania [58], lizard crania [59]. In the present study, we added a new dimension to methods for detecting modules, which allows covariance data of morphometric shapes to be analyzed using theoretical frameworks of network analysis. Our method consists of three steps: (1) conversion of variance-covariance matrix of morphometric data into adjacency matrix, which mathematically represents a complete graph, to draw morphological correlation network; (2) control of a threshold to set the topology level of morphological correlation network; (3) application of network analysis methods to the morphological correlation network satisfying a threshold. Although in this study the Reichardt-Bornholdt method to detect modules was used, in principle any other methods of network analysis can be applied to investigate covariance architecture of morphological shapes, though such applications to morphometric data seem to require further validation in individual cases. In the field of network theory, methods of identifying the modules from networks have been well developed [36,37]. The implementation of network theoretical approaches to morphometric data will provide further opportunities for understanding the complex adaptive traits of organisms.

**Figure 6:**
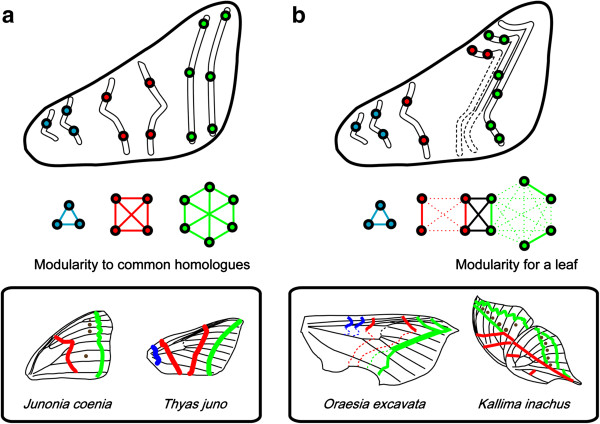
**Strategies for lepidopteran wing pattern diversification.** Schematic illustrations of divergence in moth and butterfly wing patterns. **(a)** Individualization: a commonly discussed strategy that allows the independent modification of the common (homologous) elements; **(b)** Rewiring: a novel strategy proposed in this study that allows the association of the common elements to couple and decouple to establish new modules, free from the individuality of the NGP system.

In contrast to previous reports showing that NGP symmetry elements have a higher degree of individuality during wing pattern divergence [17,24-26], our results show that modularity of NGP symmetry elements was not completely detected, but rather they were reorganized into new modules (Figure 4). This discrepancy could be attributed to the fact that the wing patterns investigated in previous morphometric studies were relatively simple and stereotypical (*Junonia coenia* and *J*. *evarete*) [21-23] (Figure 6a, bottom). In fact, consistent with previous studies, our analysis revealed that the simple wing pattern of *T*. *juno* has a high degree of modularity in the symmetry elements (Figure 5b). These results suggest that evolution toward more complex patterns such as leaf mimicry includes the coupling and decoupling processes of the constituent elements, distinct from the original stereotypical ones (Figure 6a). Previous studies have focused on the decoupling of pattern divergence; for example, dislocation and individualization of serial homologous eyespots were based on the developmental compartments [60]. In addition to these mechanisms, we propose a combination of decoupling and coupling processes that “rewires” the correlation among the common parts. In conceptual studies, Vermeij proposed a key concept, the “versatility” of a given body plan, which is evaluated by the number and range of independent parameters controlling form [61,62]. As he pointed out, the more that parameters controlling morphological structures, the greater the diversity of morphological types and the larger the potential adaptive zone. Thus, this characteristic seems to be closely associated with evolvability. Accordingly, the NGP may have a versatile property, an extraordinary flexible characteristic that allows a high degree of freedom in the modification of common elements. This “rewiring” strategy (i.e., coupling and decoupling) of the ground plan provides a new organizing principle for morphological diversification and might be applicable to complex wing patterns that have not yet been investigated (Figure 6b).

How is the NGP involved in the developmental process that establishes the modules of the leaf mimicry pattern? Clues are provided by the pattern formation of eyespots in the nymphalid butterfly *Bicyclus anynana*[24]. At the early stage, *distal*-*less* (*dll*) expression patterns are observed in all wing cells; however, as the developmental process progresses, *dll* expression disappears in the wing cells that do not form the eyespots [63]. This observation suggests that the common molecular mechanism remains in the upstream process of the developmental cascade, but also that secondary modifications in the downstream process generate a considerable difference in eyespot formation in each wing cell. Recent molecular studies have shown that the pattern elements of the NGP are formed by a common molecular mechanism (*wnt-1*, *aristaless*) in the upstream developmental mechanism [18,19]. These studies suggest the possibility that the common developmental mechanism of the NGP remains during the evolution of the *O. excavata* leaf pattern, and several modifier alleles responsible for coupling and decoupling have been fixed as secondary modifications. If so, an expression pattern similar to that of *wnt-1* may be found at the early developmental stages in both the *O*. *excavata* leaf pattern and the *T. juno* nonmimetic pattern, and subsequent expression patterns may become different to establish different modules. Testing this hypothesis will require a detailed analysis of the pattern formation processes using well-developed molecular techniques [63-70].

Although one may reasonably conclude that the leaf mimicry pattern establishes the modules, it is important to understand which factors promote the evolution of such modules. Quantitative analysis suggested that the evolution of the modules in the leaf pattern originated from the coupling and decoupling of NGP symmetry elements (Figure 5), which leads us to infer that the evolution of the correlations that established the modules is unlikely to have occurred by chance. In this respect, the conceptual idea of modularity postulates functionally related elements that are tightly correlated as modules (termed variational modules) [28,71]. This idea is consistent with the quantitative genetics perspective that variation and covariation of phenotypic traits are subject to the effects of natural selection [34,35]. One possible scenario in the evolution of the *O*. *excavata* modules is that the modules corresponding to the leaf venation components originated in response to the adaptive evolution of leaf mimesis. Additionally, this scenario may be plausible from an ecological viewpoint. Previous studies suggested that the visual appearance of mimesis appeals to the object recognition of predators, not simply the visual detection of predators [72-74], an idea that was recently validated by experimental tests using birds [75,76]. If predators are able to discriminate whether the object is edible or inedible through recognition of the morphological patterning, the patterning is hypothesized to realize a specific integration at a phenotypic level. This study cannot completely rule out the possibility that the modules are adaptive products needed for other factors (e.g., an advantage in flight or thermoregulation [77]), or merely by-products of responses to other effects (e.g., developmental constraints [78-80]). To test this hypothesis in future experiments, quantitative analysis will be useful to determine whether other lepidopteran leaf patterns show modularity corresponding to subordinate leaf-venation components, perhaps together with predation experiments using birds [81].

## Conclusions

A comparative morphological analysis dissected the leaf mimicry pattern of a noctuid moth, *O*. *excavata*, into an evolutionarily common set of pattern elements illustrated by the NGP. We developed a new method, “morphological correlation networks,” to detect the modules of the leaf mimicry pattern of *O*. *excavata* and found that the modules were established by coupling different symmetry systems of the NGP and decoupling a single element into separately correlated units. Moreover, these modules were closely associated with its leaf-venation pattern, not with the individuality of the NGP. In contrast, the nonmimetic pattern of *T*. *juno* had modules that regularly corresponded to the symmetry elements of the NGP. These results suggest that the evolution of the leaf mimicry pattern entails the evolution of new modules, which may be facilitated by a versatile characteristic of the NGP symmetry system.

## Methods

### Sampling

Two noctuid moths displaying different defensive wing patterns were investigated: *O*. *excavata* (*n* = 24) with a leaf-like appearance and *T*. *juno* (*n* = 16) with crypsis camouflage. *O*. *excavata* was established from about 10 gravid females collected at Mt. Rokko in Japan in 2006. *O*. *excavata* individuals were reared under carefully controlled environmental conditions (26°C, 80% humidity and a light/dark photoperiod of 16/8 h) in the breeding laboratory. Therefore, external environmental effects could not have contributed to individual differences. For this analysis, the *O*. *excavata* moths were maintained to the sixth generation and measured. The analyses of *T*. *juno* were based on dry specimens prepared shortly after their collection at Mt. Rokko in 2005. Unlike *O*. *excavata*, their individual differences might reflect external environmental effects. These moths were obtained conforming to local ethical regulations regarding field studies and complying with the Convention on Biological Diversity and the Convention on the Trade in Endangered Species of Wild Fauna and Flora.

### Sufficiency in the sample size

To estimate modularity in wing patterns, sufficient numbers of individuals must be sampled to ensure accurate estimations of trait correlation or covariance matrices. At low sample sizes, such matrices may become unstable, and stochastic differences among individuals can strongly affect the matrix structure. To assess the relationship between sample size and matrix stability in the wing patterns, the modular architectures were also examined using reduced sample numbers and the results were compared to those obtained using all samples. Based on these analysises, we investigated the modular architectures using the reduced numbers (*O*. *excavata*, *n* = 23; *T*. *juno*, *n* = 15), which were one less than the full sample size. In this analysis, reduction of the sample size was not quite as sensitive to fluctuations in matrix structure. The analysis was conducted in all (n-1) combinations (not only a few ones). Although the results should be interpreted cautiously, they serve as a useful approximation of the sufficient sample size appropriate for modularity analysis.

### Data

The data concerning the dorsal right forewings of both species were derived from male moths. Therefore, sexual dimorphism did not contribute to the morphological variation measured. The wings were carefully removed, mounted on slides, and digitized with a VHX-600 digital microscope (Keyence Corp., Osaka, Japan) under carefully controlled light conditions. The VHX-600 has a high-resolution accuracy of 4.5 μm/pixel. The landmarks measured (designated “reference landmarks”) were located at either the wing-vein junctions or the vein-margin intersections on the *O*. *excavata* (21 landmarks; Additional file 1a, upper wing) and the *T*. *juno* wings (18 landmarks; Additional file 1b, upper wing). Other landmarks (designated “measurement points”) were measured and located at the intersections of the wing pattern and wing veins on the *O*. *excavata* wing (19 landmarks; Additional file 1a, lower wing) and the *T*. *juno* wing (16 landmarks; Additional file 1b, lower wing). Measurement points were chosen in regions where the colors of the elements did not blend with the background.

### Procrustes superimposition

The variations and covariations were examined using standard geometric morphometrics based on a least-squares Procrustes fit [82,83]. The Procrustes superimposition consists of three successive steps. (1) Scaling: all configurations are scaled to a unit centroid size (i.e., the square root of the sum of the squared distances from each landmark to the centroid of the configuration) by dividing all the coordinates by the corresponding centroid size. (2) Translation: the centroids (centers of gravity) of the configurations are superimposed onto each other by translation. (3) Rotation: the configurations are rotated around their centroids to minimize the sum of the squared distances between the corresponding landmarks and to optimize the superimposition. The measurement points were scaled, translated, and rotated according to the information from the Procrustes superimposition conducted using the reference landmarks. This two-step procedure seems to be suitable to alleviate spurious covariance among the measurement points induced by the Procrustes superimposition, because the configuration of wing veins is more stable among individuals than that of wing patterns. This type of procedure is often used (e.g., [84,85]). The new coordinates (Procrustes coordinates) were used as two-shape variables (*x* and *y* coordinates). These procedures were conducted using the package “shapes” in R.

### Procrustes analysis of variance (ANOVA)

To estimate the amount of measurement error resulting from digitization, replicate measurements were made on a set of 24 *O*. *excavata* individuals and 16 *T*. *juno* individuals. To assess the precision of the digitization, the landmarks were dotted twice on all images. A Procrustes ANOVA [83] was performed. Because the variational modularity was assessed by investigating the covariations among pattern elements, we needed to ensure that measurement errors arising from the digitization process were negligible compared with the shape variations in the pattern elements. This was the case, because the mean square values for the individuals significantly exceeded the mean squares of the error terms (Additional file 5).

### Assessment of correlation using the Rv coefficient

To quantify the correlation among the constituent parts of the wing pattern, the Rv coefficients [86,87] were calculated between two measurement points in every possible combination. This statistical analysis was suitable for examining the covariance information of morphological shapes because it measured two sets of multidimensional variables, including measurement points on wing patterns digitized as two-dimensional variables (*x* and *y* coordinates). In addition, the Rv coefficient is recommended for geometric morphometrical analysis because it is invariant under the Procrustes superimposition procedures [55,88]. In mathematical representation, the Rv coefficient is a squared cosine between (positive semi-definite) matrices, which is a multivariate generalization of the Pearson product-moment correlation coefficient [89,90]. Although recent studies on morphological shapes have applied this statistical analysis to evaluate the correlated units between two sets of several landmarks [80,91,92], this study used this coefficient to quantify the correlation between two landmarks in every possible combination. For this study, the definition of the Rv coefficient was the correlation between two landmarks each consisting of *x* and *y* variables in two dimensions (for more general explanations, see references [86-90]). In this case, the Rv coefficient is represented as follows:

(1) $$\mathrm{Rv}=\frac{\text{trace}\;\left\{X_{i}X_{i}^{T}X_{j}X_{j}^{T}\right\}}{\sqrt{\text{trace}\;\left\{X_{i}X_{i}^{T}X_{i}X_{i}^{T}\right\}\times \text{trace}\;\left\{X_{j}X_{j}^{T}X_{j}X_{j}^{T}\right\}}}$$

where ***Xi*** ∈ {*i* = 1, 2,…, *p*} denotes a random vector that consists of two rows (*x* and *y* coordinates in the landmark *i*) and N columns (N, the number of moth samples), and ***Rvij*** denotes a symmetrical matrix of the Rv coefficient between the landmarks *i* and *j* (i.e., *rij* = *rji*, and *rii* = 1). The trace of a square matrix is the sum of its diagonal elements. Consequently, the Rv coefficient estimates the strength of association between two landmarks by quantifying the amount of inter-subset covariation normalized by the amount of intra-subset variation and covariation. The Rv coefficient is represented by values between 0 and 1. The value of the Rv coefficient is 0 if the two sets of variables are completely uncorrelated. These procedures were conducted using the package “FactoMineR” in R.

### Morphological correlation network

The observed correlation among the constituent parts of the wing patterns was visualized as a network representation (morphological correlation network). Correlation network formulations [38,39] have been explicitly or implicitly employed in various studies on metabolomics [38-40], neurodynamics [41], and transcriptomes based on gene co-expression [42,43]. In the present study, this formulation was applied to the correlated relationships among the constituent parts of morphological shapes. The correlation matrix ***Rvij*** was converted to a weighted adjacent matrix ***Wij***, in which *wij* = *rij* if an Rv coefficient satisfies a given threshold; if not, then *wij* = 0. The resulting network is a complete graph if the analyses used no threshold. The fact that ***Rvij*** is a symmetrical matrix implies that ***Wij*** is also a symmetrical matrix, logically indicating that the morphological correlation network is an undirected graph. The resulting network is therefore represented such that nodes are given by the measurement points on the wing patterns and their links depend on whether two measurement points are correlated with satisfying a given threshold. In the present study, the threshold level was set using a specific Rv coefficient value (Rv coeff.=0.2). It is not suitable to set the threshold level using a significance level (e.g., α = 0.05), because the probability of making at least one type I error rises rapidly as the number of tests increases when more than one correlation coefficient is tested for significance in an individual study [93,94].

### Sensitivity test of detected modules associated with the choice of threshold

Since the topology of the correlation network largely depends on the choice of threshold level, the modules detected using this method can be sensitive to the choice of threshold. To examine for this sensitivity, whether the modules detected from the correlation network (Rv coeff. threshold level = 0.2) could be changed with respect to changes in threshold levels (Rv coeff. threshold level = 0.4 and no threshold) was tested.

### Extraction of modules using network analyses

The RB method was employed [46] to extract the modular architecture from the correlation networks using the topology of the network and the weights of the links in the network. This method is applied based on statistical mechanics using the spin-glass model, a multi-body system consisting of multiple elements (named as spins) and their interaction with each other [95]. In statistical physics, this model is used to solve the global optimization of a given function derived from the spin-to-spin interaction systems, which results in a good approximation in a large search space by reaching the minimal state of the spins. Using the RB method with the spin states being the module indices, the modular architecture of the network is extracted by seeking the spin configuration that minimizes the energy of the spin glass. In general, modules are understood as groups of densely interconnected nodes that are only sparsely connected with the rest of the network. This method partitions the nodes into modules that minimize a quality function (“energy”):

(2) $$H(\{\sigma \})=-\sum_{i\neq j}\left(W_{\mathrm{ij}}-p_{\mathrm{ij}}\right)\;\delta \;\left(\sigma_{i},\sigma_{j}\right)$$

where ***Wij*** denotes the weighted adjacency matrix of the network calculated above; if the network analyzed is not weighted, ***Wij*** is replaced with ***Aij***. ***pij*** denotes the edge probability between node ***i*** and ***j*** according to the null model. The null model should reflect the connection probability between nodes in a network having no apparent module (i.e., community structure) [46]. In this study, the random graph (i.e., Erdos-Renyi network) was used, which is recommended by the original paper [46]. The random graph is a network where every link equally probable with probability ***pij*** = p with the same number of the edges in the network we investigated. ***σi*** ∈ {1,2,…,q} is a parameter automatically provided by a program. In the computer algorithm, it denotes the spin state (i.e., the number of modules) of node ***i*** in the graph, and the number of spin states determines the maximum number of groups allowed, which is as large as the number of nodes in the network. These analyses were conducted using the package “igraph” in R.

Although module extraction is exerted on an algorithm for searching the minimal energy state of a given function, it is possible to find several different solutions with each frequency, because a spin glass frequently includes several energy states near the smallest level. Thus, a calculation often finds a local optimum of the energy state, and repeated trials can thus reveal multiple solutions dependent on the shape of the energy landscape. From the point of view of seeking modules, these energy states (i.e., the smallest energy states and the local optima) could be detected as multiple solutions. These multiple solutions seem to represent the complexity of the covariance architecture for finding a unique module. For seeking the minimal energy state of a spin-glass, simulated annealing [96] was employed. Note that these repeated trials used the same data set of Rv coefficients, but the default values assigning the spin state (***σi***) as the module indices were changed in each of the trials.

### Availability of supporting data

The data sets supporting the results of this article are included within the article and its Additional files.

## Abbreviations

NGP: Nymphalid ground plan; RB method: Reichardt-Bornholdt method.

## Competing interests

The author declares that he has no competing interests.

## Supplementary Material

Additional file 1 **Locations of the landmarks on the moth wing. (a)** Landmarks on the *O*. *excavata* wing: 21 reference landmarks of the wing veins (upper) and 19 measurement points of the wing pattern (lower). **(b)** Landmarks on the *T*. *juno* wing: 18 reference landmarks of the wing veins (upper) and 16 measurement points of the wing pattern (lower). Click here for file

Additional file 2 **The lepidopteran wing pattern displays an orderly array of pigment cells.** Moth and butterfly wing patterns are established on the basis of pigment cells arrayed in an orderly manner **(a-d)**. **(a)** Image of a portion of a leafy wing. **(b)** Close-up of the main vein of the leaf-like venation showing the pigment cells comprising it. **(c, d)** Scanning electron microscope images of pigment and socket cells. **(c)** The flat projections are the pigment cells; the socket cells are evident as small surface protrusions and are the insertion points for the pigment cells. **(d)** Arrangement of socket cells. The sizes are indicated by bars. Click here for file

Additional file 3 **Correlation matrix of the measurement points on the *O. excavata *wing pattern.** Rv coefficients between the measurement points (MP) set on the *O*. *excavata* wing pattern in all possible combinations were calculated and listed in the lower diagonal matrix. The corresponding Rv coefficients above the threshold (Rv coefficients = 0.2) are represented in bold. Click here for file

Additional file 4 **Different frequency solutions of modular architectures detected from the correlation network of *O. excavata *wing pattern.** The correlation networks of *O*. *excavata* wing pattern were obtained according to several threshold (no threshold, **a-d**; threshold level of the Rv coefficient = 0.2, **e-h**; threshold level of the Rv coefficient = 0.4,** i**). In 10,000 trials, several modular architectures were detected and shown with the occurrence frequencies in decreasing order of frequency (the most frequent solutions, **a, e, i**; the most second ones, **b, f, j**; the most third ones, c, g; the most forth ones, **d, h**). Modules detected are represented in light blue areas. (in set) The locations of the measurement points on *O*. *excavata* wing are shown. Click here for file

Additional file 5 Analysis of measurement errors using Procrustes ANOVA. Click here for file
