# Supplementary material for: Modularity of a leaf moth-wing pattern and a versatile characteristic of the wing-pattern ground plan
Source: BMC Evol Biol. 2013 Jul 27;13:158. doi: 10.1186/1471-2148-13-158 (PMC3733769; doi:10.1186/1471-2148-13-158)
Supplement: Additional file 1 — Locations of the landmarks on the moth wing. (a) Landmarks on the O. excavata wing: 21 reference landmarks of the wing veins (upper) and 19 measurement points of the wing pattern (lower). (b) Landmarks on the T. juno wing: 18 reference landmarks of the wing veins (upper) and 16 measurement points of the wing pattern (lower). [file 1471-2148-13-158-S1.doc]

**Additional File 1 | Locations of the landmarks on the moth wing.** (a) Landmarks on the *O.* *excavata* wing: 21 reference landmarks of the wing veins (upper) and 19 measurement points of the wing pattern (lower). (b) Landmarks on the *T. juno* wing: 18 reference landmarks of the wing veins (upper) and 16 measurement points of the wing pattern (lower).
