# Supplementary material for: Modularity of a leaf moth-wing pattern and a versatile characteristic of the wing-pattern ground plan
Source: BMC Evol Biol. 2013 Jul 27;13:158. doi: 10.1186/1471-2148-13-158 (PMC3733769; doi:10.1186/1471-2148-13-158)
Supplement: Additional file 2 — The lepidopteran wing pattern displays an orderly array of pigment cells. Moth and butterfly wing patterns are established on the basis of pigment cells arrayed in an orderly manner (a-d). (a) Image of a portion of a leafy wing. (b) Close-up of the main vein of the leaf-like venation showing the pigment cells comprising it. (c, d) Scanning electron microscope images of pigment and socket cells. (c) The flat projections are the pigment cells; the socket cells are evident as small surface protrusions and are the insertion points for the pigment cells. (d) Arrangement of socket cells. The sizes are indicated by bars. [file 1471-2148-13-158-S2.doc]

**Additional File 2 | The lepidopteran wing pattern displays an orderly array of pigment cells.** Moth and butterfly wing patterns are established on the basis of pigment cells arrayed in an orderly manner (a-d). (a) Image of a portion of a leafy wing. (b) Close-up of the main vein of the leaf-like venation showing the pigment cells comprising it. (c, d) Scanning electron microscope images of pigment and socket cells. (c) The flat projections are the pigment cells; the socket cells are evident as small surface protrusions and are the insertion points for the pigment cells. (d) Arrangement of socket cells. The sizes are indicated by bars.
