# Supplementary material for: Modularity of a leaf moth-wing pattern and a versatile characteristic of the wing-pattern ground plan
Source: BMC Evol Biol. 2013 Jul 27;13:158. doi: 10.1186/1471-2148-13-158 (PMC3733769; doi:10.1186/1471-2148-13-158)
Supplement: Additional file 4 — Different frequency solutions of modular architectures detected from the correlation network of O. excavata wing pattern. The correlation networks of O. excavata wing pattern were obtained according to several threshold (no threshold, a-d; threshold level of the Rv coefficient = 0.2, e-h; threshold level of the Rv coefficient = 0.4, i). In 10,000 trials, several modular architectures were detected and shown with the occurrence frequencies in decreasing order of frequency (the most frequent solutions, a, e, i; the most second ones, b, f, j; the most third ones, c, g; the most forth ones, d, h). Modules detected are represented in light blue areas. (in set) The locations of the measurement points on O. excavata wing are shown. [file 1471-2148-13-158-S4.doc]

**Additional File 4 | Different frequency solutions of modular architectures detected from the correlation network of *O. excavata* wing pattern.** The correlation networks of *O.* *excavata* wing pattern were obtained according to several threshold (no threshold, a-d; threshold level of the Rv coefficient = 0.2, e-h; threshold level of the Rv coefficient = 0.4, i). In 10,000 trials, several modular architectures were detected and shown with the occurrence frequencies in decreasing order of frequency (the most frequent solutions, a, e, i; the most second ones, b, f, j; the most third ones, c, g; the most forth ones, d, h). Modules detected are represented in light blue areas. (in set) The locations of the measurement points on *O.**excavata* wing are shown.
