# Supplementary figures and images for: Modularity of a leaf moth-wing pattern and a versatile characteristic of the wing-pattern ground plan
Source: BMC Evol Biol. 2013 Jul 27;13:158. doi: 10.1186/1471-2148-13-158 (PMC3733769; doi:10.1186/1471-2148-13-158)

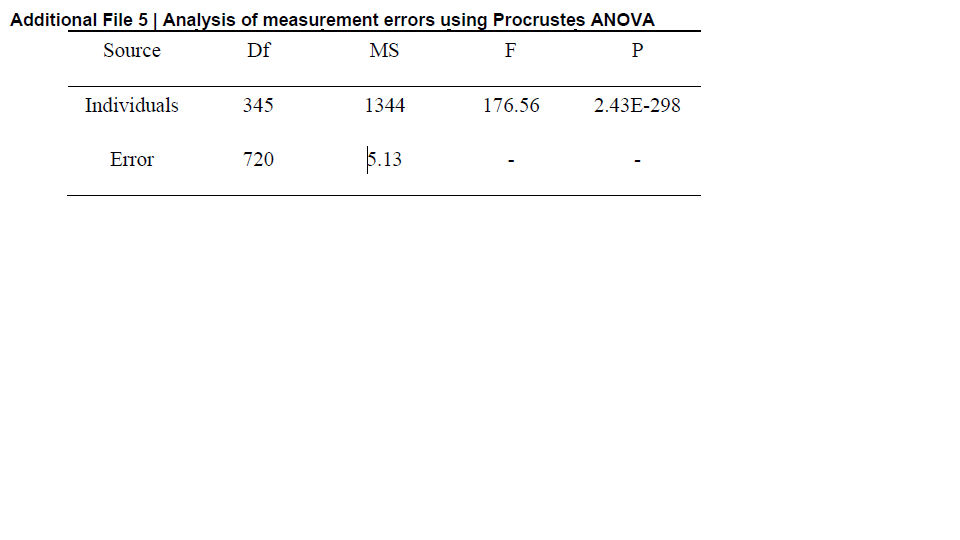

Supplement: Additional file 5 — Analysis of measurement errors using Procrustes ANOVA. [file 1471-2148-13-158-S5.png]
